# Migration experiences, life conditions, and drug use practices of Russian-speaking drug users who live in Paris: a mixed-method analysis from the ANRS-Coquelicot study

**DOI:** 10.1186/s12954-020-00398-9

**Published:** 2020-08-10

**Authors:** Yaël Tibi-Lévy, Daria Serebryakova, Marie Jauffret-Roustide, ANRS-Coquelicot Study Group, F. Barin, S. Brunet, S. Chevaliez, M. Jauffret-Roustide, L. Léon, Y. Le Strat, J.-M. Pawlotsky, J. Pillonel, C. Semaille, C. Sommen, A. Soulier, D. Thierry, L. Weill-Barillet

**Affiliations:** 1 Cermes3, Centre de recherche médecine, sciences, santé, santé mentale, société (Inserm U988/CNRS UMR 8211/EHESS/Université Paris Descartes), Campus CNRS - 7, rue Guy Moquet, 94801 Villejuif Cédex, France; 2 Santé Publique France, Saint-Maurice, France; 3 Institut Convergences Migrations, Campus Condorcet, Aubervilliers, France; 4 Baldy Center for Law and Social Policy, Buffalo University of Social Sciences, New York, USA

**Keywords:** People who inject drugs (PWID), Harm reduction, Public policy, Migration, Eastern Europe, France

## Abstract

**Background:**

After the collapse of the Soviet Union at the beginning of the 1990s, people who inject drugs spiked in Eastern Europe. Facing local repression and an array of factors encouraging emigration, some users have migrated to France. This population now make up to a third of the patient list of some harm reduction services in Paris. This article aims to present original data on the sociodemographic profiles of these users, on their migration trajectory, their life conditions, and on the evolution of their drug use practices since arriving in Paris.

**Methods:**

Data were collected as part of the ANRS-Coquelicot Survey, an HIV and HCV seroprevalence study among French-speaking people who use drugs. A sub-sample of Russian-speaking drug users who had relocated from Eastern Europe to live in Paris completed a quantitative questionnaire (*N* = 150) and a qualitative semi-structured interview (*N* = 20). The survey aimed to describe participants’ backgrounds, and a thematic analysis of interviews was conducted to explore participants’ migration histories, their life conditions in Paris, and their drug use practices before and after arriving in France.

**Results:**

This study highlights the great vulnerability of the participating population, often following a loss of social status after migrating to France. Another important finding is that participants had better access to harm reduction tools and reduced their risk of exposure to HIV and HCV infections linked to needle sharing. Although 60% said they had already shared a syringe in their lifetime (49.9% of them in their home country), the proportions shrank to 13.9% after they arrived in France and to 9.3% in the month before the study, a proportion that is lower than among French-speaking people who use drugs.

**Conclusions:**

Our main findings on the profiles and behaviors of the study population lead us to make two recommendations: to offer stronger global care to these users in Paris and to reform drug policy in their home countries by integrating it into a public health approach.

## Background and aims

The global population of people who inject drugs (PWID) is estimated between 10.6 million (8.3 to 14.7 million) [1] and 15.6 million (10.2 to 23.7 million) [2]. With a proportion of PWID between 17 [1] and 19% [2] and an injection prevalence 3.8 higher than the global average (0.82% vs 0.22%) [1], Eastern Europe is the region most impacted by this practice. Although the Russian Federation features the highest number of cases in the world [1], small countries like Georgia also feature high levels of injection practices (0.91% of PWID—almost 1 in a 100 residents) [3]. This situation, which has been noted since the collapse of the USSR at the beginning of the 1990s, can be attributed to a number of factors: higher levels of corruption in all ex-Soviet countries where the state has less power (which encourages trafficking), good perceptions of injection as projecting a Western lifestyle, quick degradation of traditional social support systems (which may have encouraged production and trafficking of illegal substances), and the sudden opening of borders (which has made the region into a prime trafficking zone between Western Europe, central Asia, Japan, and America) [4]. Although younger generations sometimes describe drug use practices in the 1990s as encouraging social relations, these have become more individual as family ties have weakened and values shifted, which Rhodes describes as a “cultural transition” [5].

The desired response to growing global drug trafficking has triggered lively debate everywhere between those who favor repression and those who defend health interventions aiming to reduce harm. Many authors have described the context in which authoritarian and repressive policies in Eastern Europe have led to immediate arrests and long prison sentences, which hinders drug users’ access to healthcare [6, 7]. Even as harm reduction services have started to develop in Georgia [3, 8] and as attempts at humanizing treatment sometimes see the light of day in Russia [9], psychoactive substance use continues to be considered a crime and to be punished as such [6]. Georgian president Mikhaïl Saakachvili’s anti-drug policy and anti-corruption policies, enforced between 2004 and 2013, are a prime example of this: arbitrary urine tests in the population, high fines, and prison sentences [8]. On one side, it has become clear that repression, enforced for the last 15 years in Georgia, has not only failed to eradicate drug use but has also contributed to higher expositions to risk for drug users by encouraging risky practices and exposure to HIV and hepatitis C transmission (sharing equipment, homemade injectable products) [10]. On the side, international research has shown that policies based on mass incarceration of PWID are ineffective for the diminution of drug use and are counter-productive as they contribute to the spread of infectious diseases in an already vulnerable population [1, 6, 11]. Hepatitis C and HIV prevalence among PWID are therefore significantly higher in Easter Europe than in the rest of the world (64.7% vs 52.3% for hepatitis C, 24.7% vs 17.8% for HIV) [2].

Repression of this kind, along with health, family, economic, ethnic, religious, and/or geopolitical factors, is one of the main factors influencing many Eastern European PWID to migrate westward [3]. In fact, France (particularly Paris) has witnessed significant migrations of PWID coming from these countries, mainly from the Russian Federation, Georgia, Ukraine, and Armenia [12]. Eastern European PWID now make up to a third of the patient list of some Harm Reduction Facilities (called Centre d’Accueil et d’Accompagnement à la Réduction des Risques pour Usagers de Drogues—CAARUD), which offer access to clean injection equipment and to opioid substitution treatment) in the Paris area. The majority of them are from Georgia [13], probably due to cultural, diplomatic, and economic ties that have existed between France and Georgia for many decades.

This type of immigration has also led some French clinicians to seek out the specific characteristics of Georgian PWID and to understand the anti-drug policies enforced in their home countries. Some have defined this situation as a “drug use epidemic,” noting that drug use has permeated all types of social environments [12]. They also highlight the deleterious effects of these policies, which they say favor “social vulnerability, suffering, and black-market economies” [14]. When they arrive in France, this population is often at the receiving end of powerful stigmatization inside addiction care centers and harm reduction services—stigmatization that stems from both French-speaking PWID and from harm reduction professionals [12]. Eastern European users are often poorly perceived. They are often presented as belonging to the Mafia and/or as disinclined to take into account harm reduction measures proposed in France, particularly when it comes to using clean injection equipment. This negative prejudice often comes with attention from the media, which tends to circulate age-old fears about the resurgence of multi-resistant tuberculosis and more generally about infectious diseases [13].

The issue of PWID migration from Eastern Europe, particularly from ex-Soviet countries, has not yet been studied in France. In this context, we implemented the ANRS-Coquelicot Study that included Russian-speaking PWUDs. This article aims to present original data on the sociodemographic profiles of these users, on their migration trajectory, their life conditions in France, and on the evolution of their drug use practices since arriving in Paris.

## Methods

### ANRS-Coquelicot for Russian speakers: a multi-centered study

Between 2013 and 2015, Inserm and Public Health France have set up an epidemiological and sociological study on Russian-speaking PWID in Paris, in collaboration with the National Reference Center (CNR) on HIV and with the CNR on hepatitis B and C, and with the financial support of the National Research Agency on HIV and hepatitis (ANRS). This study complements the HIV prevalence study (ANRS-Coquelicot) conducted in 2011 among French-speaking PWUD [15]. For this study, we used Time Location Sampling design and selected a random sample of PWUD recruited in almost all addiction centers and harm reduction facilities in Paris or Seine-Saint-Denis. This includes 70 services of three kinds: Centers for Drug Use Harm Reduction Support (Centres d’Accueil et d’Accompagnement à la Réduction des risques liés à l’Usage de Drogues—CAARUD), which includes fixed and mobile syringe exchange programs; Centers for Drug and Alcohol Addiction Healthcare (Centres de Soin, d'Accompagnement et de Prévention en Alcoologie et Addictologie—CSAPA), which includes access to opioid substitution treatments and hospital services; and shelters and housing services.

In order to find out which services had more than 30% of Russian speakers in their patient list, we created a list of services. We recruited a sample of users in these services using a two-tiered sampling design: first, we made a list of all services open on a half-day basis so as to draw at random service/half-day pairs, using simple random sampling. Then, researchers randomly recruited the first user who showed up in each service. Other users were chosen according to a sampling step adapted to the size of services so as to avoid the researcher or the facility’s professionals selecting the survey respondents, which would have created a selection bias. Sampling weights were calculated according to the Generalized Weight Share Method (GWSM) so as to account for the weight of each service, of each drug user, and of volume of attendance of each service [16, 17].

Out of 900 PWUD who participated in the ANRS-Coquelicot Study, 150 came from Eastern Europe. This subpopulation, which was mostly made up of men (97.2%), had an average age of 36.7 years (22–59 years) (Table 1). On top of epidemiological and biological data, we gathered other types of information for each individual, such as their sociodemographic profile, their migration history, their life conditions in France, and their drug use practices before and after arriving in Paris. We chose to conduct the entire investigation in Russian (it is the most commonly spoken language among migrants coming from ex-Soviet countries), which made it possible for a single research assistant to conduct the survey in its entirety (both quantitative survey and qualitative interviews). Participation criteria included having injected or snorted at least once in lifetime, being of age, and speaking Russian (though not necessarily as a primary tongue). The question grid we used, which was designed specifically for this study, was anonymous, confidential, and based on voluntary participation. The questionnaires were administrated by a professional, external research assistant trained by the principal investigator of the survey. The completion of the questionnaire lasted about 45 min, including securing oral consent. The protocol was approved by the committee for the protection of individuals in Créteil, France.

**Table 1:** Profiles of interviewees from the quantitative survey, *N*=150

	%
**Sex**	
Men	97.2
Women	2.8
**Age** (average, 36.7 years old; SD, 7.7 years; min-max, 22–59 years old)	–
< 30	13.9
30–34	31.3
35–39	24.1
40–44	12.5
45–49	9.3
50+	8.9

In order to explore certain issues that were not the focus of the quantitative section of the study and to gather new kinds of information, we created a sociological component to the study. With 20 (18 men and 2 women) of the 150 Russian-speaking individuals interviewed, we conducted semi structured interviews (Table 2). An offer to participate in an interview for the qualitative survey was introduced at the end of the quantitative survey, and when possible, an appointment was made with interested participants. All interviews were transcribed in full, translated to French, and made anonymous (all names were modified). After gathering this data, we had at our disposal a wealth of materials made up of both quantitative and qualitative information.

**Table 2:** Profiles of interviewees of the qualitative survey *N*=20

*N* user	Name (false)	Sex (H/F)	Age (in years)	Time spent in France	Place of birth	Home country
1	Alik	Man	34	3 years	Chechnya^a^	Russia^a^
2	Jino	Man	23	3 years	Georgia (Yezidi Kurd)	Poland
3	Dimitri	Man	29	1 year	Russia^a^	Russia^a^
4	Kirill	Man	27	1 year	Kazakhstan	Chechnya^a^
5	Igor	Man	38	12 years	Russia^a^	Russia^a^
6	Bakar	Man	30	5 months	Chechnya	Russia^a^
7	Youri	Man	28	2 1/2 months	Russia^a^	Russia^a^
8	Kelvin	Man	35	2 years	Georgia	Georgia
9	Donovan	Man	36	2 months	Georgia	Russia
10	Mosvar	Man	39	2 years	Ingush Republic^a^	Chechnya^a^
11	Olga	Woman	27	5 years	Russia^a^	Russia^a^
12	Ulan	Man	43	10 years	Kazakhstan	Russia^a^
13	Sarkis	Man	39	3 years	Armenia	Armenia
14	Anzor	Man	34	2 years	Chechnya^a^	Chechnya^a^
15	Austin	Man	45	3 years	Georgia	Russia^a^
16	Nikolaï	Man	39	6 years	Russia^a^	Russia^a^
17	George	Man	50	10 years	Georgia	Georgia
18	Ivan	Man	42	A few days	Georgia	Georgia
19	Lévon	Man	27	2 weeks	Armenia	Georgia
20	Monika	Woman	28	6 months	Lithuania	Lithuania

^a^Contrary to other regions cited here, Chechnya and the Ingush Republic are not autonomous states but republics of the Russian Federation claiming their right to independence

### Data analysis

We analyzed quantitative data (*N* = 150) using the Windows version of IBM SPSS 22.0 (IBM Corporation, Armonk, NY, USA) based on univariate descriptive approach and taking into account sampling weights. The database was anonymous and included sociodemographic data, reasons and trajectories of migration, life conditions in home country and in Paris, and evolution of psychoactive drug use practices since arriving in France. After selecting the most relevant variables, we described the population: average, median, standard deviation, minimum and maximum for continuous variables (age, number of years since departure from home country, duration of incarceration…), and frequencies and proportions for categorical variables (educational level, context of first injection, financial resources…).

We processed qualitative data (*N* = 20) by conducting a thematic analysis. We analyzed each interview transcription through an iterative process: after several readings, we created a codebook of interest topics (themes) in an excel file guided by the qualitative data set, using an inductive approach. This inductive approach to analysis, based entirely on raw field data, is part of our effort not to be influenced by preconceived hypotheses or established frameworks so that we could deliberately produce an analysis anchored in empirical data, thereby reflecting most subtly the lived experiences of the study participants. After this thematic classification, we listed all the arguments cited into a double entry table: we inserted the 20 interviewed drug users into the row heads and inserted the way their answers contributed to our understanding of each topic in the column heads. This table, which can be read both horizontally (by person interviewed) and vertically (thematically), became our starting point to analyze contents. Following a thematic analysis, quotes from the interviews were chosen to highlight themes.

We set up a process of triangulation between our quantitative and qualitative research data: with the quantitative elements, we were able to describe the profiles and practices of Russian-speaking PWUD who attend health and harm reduction facilities in Paris by using the same sampling design that was already in use in the study. This allowed us to create representative samples for this population. With the qualitative elements, we were able to hone in on issues brought up in the quantitative data by creating more nuanced descriptions of practices and social profiles based on a more limited number of participants. The qualitative component of our research made clearer the roles of various social processes (war, repression, political regime) in shaping East-to-West migration patterns, as well as the impacts of migration on use practices and risk exposure. The qualitative component also made it possible to adopt a longitudinal approach to Russian-speaking PWUD’s life trajectories, which was not possible in the quantitative study, a transversal, punctual form of data gathering that does not give information about life histories. The two components of our research were thus complementary and made it possible to create a precise, nuanced, and in-depth view of Russian-speaking PWUD who live in France.

## Results

Our main results are presented in Tables 3, 4, 5, and 6.

**Table 3:** Origin and reasons for migrating, *N*=150

	%
**Place of origin**	
** Birthplace**	
Georgia	55.0
Russia (outside Chechnya and the Ingush Republic)^a^	16.3
Chechnya^a^	10.2
Lithuania	7.6
Others (Ukraine, Kazakhstan, Armenia, Latvia, Tajikistan, Ingush Republic^a^, Belarus…)	10.8
** Country of origin**	
Georgia	57.2
Russia (outside Chechnya and the Ingush Republic)^a^	16.1
Chechnya^a^	9.7
Lithuania	7.6
Others (Ukraine, Kazakhstan, Armenia, Latvia, Tajikistan, Ingush Republic^a^, Belarus)	9.4
**Migration trajectories**	
Number of years since departure from home country (avg, 5.3 years; SD, 5.9 years; 0–24 years)	
0 (less than a year)	9.7
1–3 years	45.5
4+ years	44.8
**Number of registered countries of residents (avg, 1.9 countries; SD, 1–2 countries, 1–10 countries)**	
1 country	41.3
2 countries	40.0
3+ countries (max = 5, then 10)	18.7
** Countries of residence for 1 year or longer**	
Russian speaking ex-Soviet countries	40.7
Other Eastern European countries	9.6
Western European countries	20.5
Other countries	8.3
**Five most recurring countries of residence**	
Russia	36.5
Germany	9.6
Poland	9.2
Ukraine	5.9
Spain	5.2
Other: 1/Belarus, Slovenia, Latvia, Ingush Republic, Kazakhstan, Armenia, Bulgaria, Austria, Czech Republic; 2/UK, Netherlands, Belgium, Luxembourg, Switzerland, Sweden, Denmark, Portugal, Italy, Israel, Turkey, Tunisia; 3/USA, Canada, India	
**Arrival in France**	
Number of years since arrival in France (avg, 3.3 years; SD, 4.1 years, 0–21 years)	
0 (less than 1 year)	21.5
1–3 years	48.8
4+ years	29.7
** Arrived in France with whom?**	
Alone	61.3
Friends/countrymen	5.6
Family	33.1

^a^Contrary to other regions cited here, Chechnya and the Ingush Republic are not autonomous states but republics of the Russian Federation claiming their right to independence

**Table 4:** Drug use initiation and evolution of products consumed, *N*=150

	%	
** Countries where drugs were used**		
In home country AND in France	72.2	
In home country AND only as substitution treatment in France	4.8	
In France AND never in home country	19.5	
Only abroad (neither in home country nor in France)	3.5	
** Country of first drug use**		
Home country	77.0	
France	19.5	
Other	3.5	
**First injection**		
Injection at least once in lifetime		
No	4.5	
Yes	95.5	
** Age of first injection** **(avg, 21.8 years old; SD, 6.2 years, 13–40 years old)**		
1 < 20	41.0	42.9
2 20–24	30.3	31.7
3 25–29	13.5	14.2
4 30+	10.7	11.2
N/A (never injected)	4.5	–
** Context of 1st injection**		
Alone	26.0	27.2
With assistance of third party	69.5	72.8
N/A (never injected)	4.5	–
** First product injected**		
Heroin	41.7	43.6
Cherniashka (opioid concoction)	16.9	17.7
Morphine	10.3	10.8
Opium	7.9	8.2
Buprenorphine (Subutex)	6.3	6.5
Other: morphine sulfates (Skenan, Moscontin), desomorphine, krokodil, benzodiazepines, cocaine, tranquilizers, vint	12.5	13.1
N/A (never injected)	4.5	–
**Evolution of products consumed since arrival in France**		
**Injection in the last month**		
At least one injection in the last month	71.1	
Daily injection in the last month	50.4	
Weekly injection in the last month	30.7	
Monthly injection in the last month	18.9	
**Products consumed in home country**		
Cherniashka (opioid concoction)	46.7	
Heroin	21.3	
Pervitine, ephedrine, vint, jeff	14.1	
Crystal meth	9.4	
Desomorphine, krokodil	5.2	
Buprenorphine (Subutex, Temgesic)	3.4	
Opium	2.3	
Cocaine	1.6	
Amphetamines—speed	1.3	
Codein, morphine	2.6	
Other (brown sugar, pills/medication)	0.4	
**Products consumed in France**		
Morphine sulfates (Skenan, Moscontin)	54.4	
Methadone	37.8	
Buprenorphine (Subutex, Temgesic)	33.3	
Cocaine	24.5	
Crack, free base	15.0	
Amphetamines—speed	2.3	
Heroin	2.2	
Opium	0.4	
** Non-Adherence to harm reduction recommendations (syringe sharing)**		
Syringe sharing in the last month	9.3	
Syringe sharing since arrival in France	13.9	
Syringe sharing in home country	49.9	
Syringe sharing in lifetime	62.3

**Table 5:** Family, employment, and prison, *N*=150

	%	
**Living at home with family at age 16**		
No (single/in a relationship, school/boarding, prison, at someone else’s house)	6.6	
Yes (with 2 parents, single father, single mother, other parents)	93.4	
** Living at home with family at age 16 (with details)**		
With 2 parents	74.2	79.5
With single mother or single father	15.5	16.7
With another parent	3.6	3.8
N/A (not living at home)	6.6	–
**Public housing or living on the street since age 18**		
In public housing (yes, once or multiple times)	72.7	
On the street (yes, once or multiple times)	79.7	
**Relationship status at time of study**		
Are you in a relationship?		
Single	39.1	
In a relationship	60.9	
**Living with a partner**		
No, never have	14.1	23.2
Yes, at times	8.6	14.1
Yes, always	38.1	62.7
N/A (single)	39.1	–
**Education level**		
Primary	4.3	
Secondary (middle school, high school, and technical degrees)	52.7	
Higher (including military school)	43.0	
**Employment situation in France**		
Are you employed?		
No	96.8	
Yes	3.2	
**Employment**		
On benefits	16.2	16.7
Invalid	0.3	0.3
Not authorized	80.4	83.1
N/A (employed)	3.2	–
**Prison experience**		
Time in prison		
No	42.4	
Yes	57.6	
**Country of incarceration**		
Prison time in France	16.6	28.7
Prison time in country of origin	34.4	59.7
Prison time in another country	20.5	35.5
N/A (no prison time)	42.4	–
** Number of times incarcerated**		
1	22.2	38.5
2 to 5	31.8	55.3
6 or more	3.6	6.2
N/A (no prison time)	42.4	–
**Duration of incarceration (avg, 3.4 years; SD, 3.0 years; 1 month–15 years)**		
1 to 5 months	9.4	16.4
6 to 11 months	9.8	17.0
12 months to 5 years	22.0	38.1
6 to 9 years	14.6	25.4
10 to 15 years	1.7	3.0
Do not know	0.1	0.1
N/A (no prison time)	42.4	–
** Injection while in prison**		
No	35.4	61.5
Yes	22.2	38.5
N/A (no prison time)	42.4	–

**Table 6:** Life conditions in France, *N*=150

	%	
** French language proficiency**		
No	37.6	
Rudimentary	41.4	
Autonomous	20.9	
** Access to French language assistance**		
No	33.4	42.2
Yes, sometimes	13.6	17.2
Yes, always	31.0	39.2
No answer	1.1	1.4
N/A (French proficiency)	20.9	–
**Financial resources**		
Employment (odd jobs)	35.0	36.2
Pensions, allowance, donations from associations	22.3	23.0
Financial assistance from friends/family	46.1	47.6
Begging	5.6	5.8
Theft and drug dealing	17.2	17.7
Unemployment, street donations, prostitution	0.0	0.0
Other (sold apartment in home country)	0.1	0.1
No resources	9.8	–
**Type of housing**		
Housing type (at time of investigation)		
Stable (with a partner or homeowner…)	10.0	
Precarious (social housing, friends/family, hotel room paid by own expenses…)	61.4	
Highly precarious (squat, public space, vehicle…)	28.6	
** Housing (for the coming night)**		
At the home of third party (partner, family, friend, countryman)	14.7	
Personal housing (owned or rented housing, independent hotel)	16.2	
Drug user housing (post cure, therapeutic housing, public housing center)	1.4	
General housing (associations, collective housing, association hotel)	39.1	
Space not designed for housing (squat, public space, vehicle)	28.6	

### Migration trajectory (Table 3)

#### Origins

The majority of survey respondents were born in Georgia (55.0%) and to a lesser extent in Russia (16.3%), Chechnya (10.2%), and Lithuania (7.6%). The others, who represent a minority, were born in Ukraine, Kazakhstan, Armenia, Latvia, Tajikistan, the Ingush Republic, or Belarus. This distribution is the same when considering the place of origin and not of birth, meaning the place where they lived before emigrating (57.2% of Georgian residents). Although respondents have lived in France for 3.3 years on average (from a few days to 21 years), they have left their home country 5.3 years ago on average (0 to 24 years). Based on these numbers, we estimate at 2 years the average duration of their migration trajectory before arriving in France. Respondents have crossed one or several countries between their home country and France (1.9 on average).

Half of them (50.3%) have lived at least 12 months in an East European country other than their own (Russia, Poland, Ukraine...), 20.5% in a Western European country (Germany, Spain...), and 8.3% outside Europe (the USA, Canada, India, Tunisia...). Their arrival in Paris is therefore part of the long migration trajectory, as some of them (18.7%) lived for more than a year in at least three countries before France. Interviewees migrated either legally (with passport or visa^1^) or illegally (“Since I have not received a visa, I cross the border and I walked from the Belarus-Poland border to Warsaw in 10 days. I hitchhiked to Berlin. [...] I crossed all of Germany and then all of Italy. Always hitchhiking. I walked with my backpack, and I only ate whatever those who took me on gave me,” Dimitri, 29 years old, Russia) and were sometimes required to pay significant amounts of money (“I had applied for the standard Schengen visa. €3000 for a tourist visa in two days; […] there’s an agency who does that at home. They don’t get visas to the Chechen, you see, because there is a direct presidential order not to let the Chechen leave,” Anzor, 34 years old, Chechen).

#### Migration groups

Although most migrants arrived in France on their own (61.3%), a significant portion came with their families (spouse, children, or more rarely, parents) (33.1%). Those who came with friends or other countrymen are rarer (5.6%). With therefore noted two types of migrants: (1) those who have a family who came before, at the same time, or after them and who can benefit from their support (“My brother had lived here for eight years. He helps me morally and materially. I see him often,” Alik, 34 years old, Russian born in Chechnya) (“[My spouse] is doing everything for me to stop. She’s the one who helped me stop injecting,” George, 50 years old, Georgian); and (2) those who only have more or less close acquaintances or friends and who have a harder time integrating (“My parents and my sister are in Armenia. […] I know a lot of people who live here, [but] because I have weak health, no one wants to hang out with me, no one wants to see me, no one wants to take the responsibility of helping someone with a physical disability and who can't go around on their own,” Sarkis, 39 years old, Armenian).

#### Reasons for migrating

Through our qualitative analysis, we identified four main reasons for migrating. Not mutually exclusive, they often reveal painful personal or collective family events:

Fleeing war (particularly for Chechens and Georgians) for reasons of identitarian, ethnic, or religious resistance (for Yezidi Kurds in Georgia in particular), and/or fleeing a totalitarian regime (that leaves no place to opposition) is frequently mentioned as reasons for migrating to apply for political asylum. Being sent to a warfront, being hunted down by police or the mafia, facing kidnappings, incarceration, torture, death threats, murders, blackmail, denunciations, false accusations, arsons…, there are many narratives of traumatizing daily life: (a) “I was in Chechnya from 1993 to 1994, during the first war. Then I went home and they told me […] that they would catch me as Zviad supporter. There was a political period like that, when nothing made sense. […] I was forced to flee in 2001.” (George, 50 years old, Georgian); (b) “My brother was barely 17 when he was gunned down by the Federal Army, and my father to was shot in front of our door by strangers.” (Kirill, 27 years old, Chechen born in Kazakhstan); (c) “My neighbors said I was a fighter, they circled my house and started to shoot with flamethrowers. I fled, […] they killed a worker and my sister.” (Mosvar, 39 years old, Chechen born in the Ingush Republic); (d) “[After] the Soviet Union collapsed, […] I saw a policeman assault a pregnant woman […]. I told him, ‘listen, you’re Armenian […], how can you shove a pregnant Armenian with a truncheon?’ He turned around, he insulted me, I hit him. […] When I opened my eyes, […] they told me […] that other policemen have hit me, had thrown themselves at me, and I fell into a puddle. And when I was in the puddle, one of them stuck an electroshock in my back and didn’t let go for almost a minute.” (Sarkis, 39 years old, Armenian); (e) “When they put me in prison, they injected me with what special services use to prevent people from sleeping for a week and to make them talk. So that they will say everything.” (Lévon, 27 years old, Georgian born in Armenia).

Another political reason to emigrate is to escape repressive drug policy. In Russia, Chechnya, and Poland in particular, consuming or possessing drugs is punished very harshly with heavy prison sentences, as two interviewees explained: (1) “In my country, if they catch you for having bought the syringe at the pharmacy, they just destroy you like you wouldn’t believe.” (Jino, 23 years old, Polish born in Georgia); (2) “In Chechnya, they oppress you for one joint; if three kids are caught smoking a joint, they all go to prison for four years. […] There was one guy with me, on top of the four years they gave him in the Russian Federation, […] he was sentenced for six more months […], so all in all four and a half years of rigorous imprisonment.” (Bakar, 30 years old, Russian born in Chechnya).

Another reason to leave is to seek adequate treatment, as Donovan and Jino, who came to Paris following hearsay, explain: (1) “I [came] here to get treatment because I had hepatitis. They told me: go to France they will cure everything.” (Donovan, 36 years old, Polish born in Georgia); (2) “[When I was In Poland], people told me in France was a hundred times better than here. To get treatment, and for everything else [...]. So I came.” (Jino, 23 years old, Polish born in Georgia). Sometimes parents take the initiative of sending their children to Western Europe to seek cessation treatment, as these two Russian users explain: (1) “The centers in Russia told my parents it wasn’t working. I was always relapsing. They suggested Spain. At first I didn’t want to, but then I got used to the idea: why not? I went to the Christian center in Spain.” (Youri, 28 years old, Russian); (2) “My mom told me that because I was injecting, because I was drinking, everyday I wanted to end my life… [She] makes €200 a month. Do you know how much these clinics cost where I’m from? […] Of course it was horrible to let me go to a foreign country, of course she didn’t want to. But the choice was clear: either I died there or I survived here.” (Olga, 27 years old, Russian). The aim is then to gain access to antiviral medication (for HIV and hepatitis C) and to opioid substitution treatments (methadone, buprenorphine), treatments which are not accessible in their home country due to repressive policies. In fact, their image of France is often positive or even idealized, far from what they have known in their home countries, as Jino expresses gratefully: “[France] is such a civilized country. Here they understand when someone is sick. It’s called a disease. There’s no reason to make you suffer even more, to chase you down. Here on the contrary they give you a hand and help you heal.” (Jino, 23 years old, Polish born in Georgia). In a new context in which addictive drug use is considered a disease and not only criminal behavior, they can now enjoyed certain rights and access certain forms of care: medical residence permit, State medical help (Aide Médicale d’Etat—AME), universal healthcare insurance (Couverture Maladie Universelle—CMU), and sometimes disability status and allowance by the MDPH (Maison Départementales des Personnes Handicapées).

Finding work and better life conditions is one last reason to leave, as Youri explains: “I heard many times that the work situation and France was better; that’s why I decided to come here.” (Youri, 28 years old, Russian). Some find themselves in survival conditions, like Ulan, who holds several unskilled and sometimes illegal jobs: “[After my parents died], I did some poaching in the woods. I did some fishing, […] I killed some boars. I would smoke the fish and deliver it to restaurants. It was little money, I had almost nothing to survive. […] I left for Moscow […] and that the end of the day I found myself sleeping in a train station. […] They’re all kinds of people there have to unload train cars. These are day jobs for day pays. […] Then we did a bit of gardening work, we worked in cottages where the water came out of the pipes, we dug some pits. […] Whatever I made I spent on food and accommodation. Then winter came, the season was over, and I couldn’t book hotel rooms anymore. […] [Then] I got several proposals to steal, to do some burglaries […] If my parents could see me, they would turn in their graves. I made some money stealing dogs, pedigree dogs, bullterriers, small puppies... I stole them and gave them back against a reward. I had to survive… […] [Then] I went to Spain where I sold fish, and then the season ended […] and I had nowhere to go. So I went to France. I got to Paris. It was raining, it was cold, I didn’t know anyone. I came out at Gare de Lyon. So actually, I came to France and that was all.” (Ulan, 43 years old, Russian born in Kazakhstan).

### Initiation into drug use and evolution of drug use practices (Table 4)

Three-fourths of respondents (77.0%) said they started using drugs in their home country. One in four (23.0%) said they started after migrating, and one and five (19.5%) said they started after arriving in France.

#### Context for drug use initiation

Although interviewees describe their first experience of drug use in the context of get-togethers among friends smoking cannabis to have a good time (“Several of us got together, five or six people, every night. […] we would buy a ‘ship’ of weed,” Nikolaï, 39 years old, Russian), our qualitative analysis revealed three contexts of experimentation, the first two of which are the most frequent:

In seeking social contact, many interviewees started to use drugs in the context of school. Although some of them interpreted as related to hanging out with the “wrong crowd” (“When I was a kid, I wanted to learn, but the teacher put me in the back of the room, with the bad kids […] In the beginning, we smoked little colored sticks, we were up to no good, we pretended to smoke and then we were tired of pretending, we saw how older kids smoked and we went to smoke behind the parking lot,” Dimitri, 29 years old, Russian), most interviewees said they started using in order to fit in to a social group and to try new experiences (“I grew up in that kind of group. Absolutely everyone that I knew was injecting. No one gave me anything, I just saw that they were shooting up. […] After a while, I just told myself ‘just try’,” Lévon, 27 years old, Georgian born in Armenia; “When I was young, I told myself: […] what are these drugs that make you fly. I wanted to fly to. And there you go, I flew, I went and saw for myself,” Donovan, 36 years old, Russian born in Georgia).

Traumatic experiences, which encourage interviewees to see their initiation to injection through the lens of a descent to hell, as Olga and Dimitri explain: “I lived through a tragedy. My father left us, my mother and me. […] I had one brother before me. He died of blood cancer after I was born. […] Me and my husband, we got hurt in a car accident when I was pregnant. He died, and the kid too, but I survived. I started to shoot up, to get fucked, to cut myself, to take sleeping pills, so that I could be close to them, so that I could die.” (Olga, 27 years old, Russian); “I was under heavy stress because I was going through a divorce and I lost custody of my child. […] They didn’t let me go into my apartment and according to my passport, I was homeless. […] I had no roof; I went wherever my feet took me. Which is Westward. So I got here. […] At first I smoked marijuana, I didn’t shoot up, I drink reasonable amounts of whiskey […] I only started to shoot up when I got to Nice. I tried something bad, some sort of concoction, I remember the feeling. With my teammate, we were dissolving pills, one for him and one for me.” (Dimitri, 29 years old, Russian).

In seeking relief from chronic pain, out of 20 interviewees, five said they started injecting morphine or heroin as painkillers for discomfort that would not go away (following spinal disk displacement after an accident, an angina that went untreated in prison, or even a home operation to extract a bullet) and then became dependent the product. Mosvar’s story is most revealing in that respect: “In 2004, they settled scores in Moscow. […] They started shooting at us. I got shot […] in the legs. […] I couldn’t go to hospital, because they have police monitoring people who come in after getting shot. I stayed home and I paid a nurse $2000, she came and she anesthetized me with morphine twice today, in the morning and at night. For about two weeks. Two weeks later, I was using crutches, everything was swollen, I had sores, it was gross. It was good that there was a nurse to help me, but because of her I became addicted to morphine. And when she stopped coming around, I started to feel craving,” (Mosvar, 39 years old, Chechen born in the Ingush Republic). Some former soldiers, particularly Georgians, said they received morphine injections during their military service to make some more aggressive (“When the war started in 1989-1991 […], I was wounded, I had a concussion, and the way things went, they gave all soldiers bottles of morphine. Everyone was shooting up, they gave it to keep fighting here and there. […] When you're shooting up, you think differently,” George, 50 years old, Georgian).

#### Context of first injection

As Dimitri explains, the first injection experience is seen as an event, a steppingstone in a drug user’s life story: “Smoking weed is one thing, injecting is another.” (Dimitri, 29 years old, Russian). Almost all survey respondents (95.5%) had injected at least once. Three-fourth (72.8%) said a third party helped them inject. The average age for the first injection is 21.8 years old, but the deviation is significant, going from 13 to 40 years old (median, 20.0 years old). The most common first product injected is heroin (in 43.6% of cases), followed by cherniashka^2^ (in 17.7% of cases), morphine (10.8% of cases), opium (8.2%), buprenorphine^3^ (6.5%), and other substances in marginal proportions (morphine sulfates, benzodiazepines, cocaine, tranquilizers, desomorphine^4^, vint^5^, krokodil^6^). A more in-depth analysis of our data brings to light a significant difference between the product used in first injections and the age of respondents at the time: thus, although cherniashka and morphine were more popular among 20 year olds, heroin is more present among 20 to 24 year olds, opium among 25 to 29 year olds, and buprenorphine and other medication among older users (25 to 29 year olds and older).

Although some PWID said they first tried injection because they were curious to experience this practice, wanted to try it for their own sake, or wanted to imitate others (“Everyone is shooting up so I decided to try it too,” Igor, 38 years old, Russian), others remember it as peer pressure within a friend group (“I was snorting. And everyone was mad at me. I asked them what difference it made how you took your products. [...] But they said no. They ended up convincing me and I started shooting up,” Alik, 34 years old, Russian born in Chechnya). They say that they were pushed into it either by acquaintances (“He told me, ‘Ulan, take a shoot, there’s no problem, that whole thing where you’re supposed to forget everything, that’s not true [...], don’t worry’,” Ulan, 43 years old, Russian born in Kazakhstan) or by strangers (“Two druggies, they knew [...] we had money. And they started to pick at us: come on, let’s go cook, bla bla bla. And we bit, you know. They warmed us up and then they branded us. It was clear, but I didn’t understand it,” Nikolaï, 39 years old, Russian). Some like Dimitri or Kelvin insist that their first experience of injection was either nonconsensual or accidental: “Men and women are preparing something, boiling it up. I ask what it is. [...] They tell me it’s ok, it’s nothing. ‘Just do it once, nothing will happen to you.’ At a glance, I see the needle coming towards me. Someone says ‘give me your arm,’ a girl takes my arm and puts the needle in the vein.” (Dimitri, 29 years old, Russia). “It’s always like this, [as Kelvin sums up his history of drug use] there’s a moment, a minute, a second that decides everything that will happen later. [...] I just found myself in the wrong place at the wrong time.” (Kelvin, 35 years old, Georgian).

Sometimes these first injections happen in the context of previous polydrug use practices involving cannabis, alcohol, amphetamines, and/or sleeping pills, as shown in these two interviews: “When I tried heroin for the first time, I was taking vint, I was smoking, I was drinking vodka. I was poisoning myself, cutting my veins, taking sleeping pills. Everything, actually. [...] [Drugs] sharpen your mind, heighten your sense of sight and all your sensations, put you in such a state of euphoria. But then there’s a kind of depression when you’re coming down. Many people hung themselves or tried to exit through the upstairs balcony to take a stroll. That’s hard.” (Olga, 27 years old, Russia); “I started injecting powdered methadone and chemical heroin. I became addicted to this shit in Saint Petersburg. Then to get away from it, I injected amphetamine for over two months to get out of methadone and heroin. Then to get away from amphetamine, I drank champagne and alcohol... alcohol, alcohol, always more alcohol... with marijuana.” (Dimitri, 29 years old, Russian).

#### Evolution of products consumed between home country and France

Among those who had a history of drug use in their home country, a minority (6.2%) said they stopped taking illegal products in France, like Jino: “Ok, you made a mistake. That’s human. So I came to get treated here [...] They give you a chance to overcome it [...] And since that day, I have not taken anything for two years.” (Jino, 23 years old, Polish born in Georgia). Most of those who had started using before migrating (93.8%) continue to use and particularly to inject (71.1%) in France.

Although these practices often continue after users migrate, however, we can observe some significant changes of products consumed since users arrived in Paris. First, some substances that were popular at home have been left behind (cherniashka, pervitin^7^, ephedrine^8^, vint, jeff^9^, crystal meth^10^, desomorphine, krokodil) or partly abandoned (heroin). Second, some products make an appearance (morphine sulfates, methadone, crack, free base) or a comeback (buprenorphine^11^, cocaine) into the panoply of drugs used. Since they arrived in France, more than half (54.4%) have injected morphine sulfates at least once, 4 out of 10 have injected methadone (37.8%), and one in three have injected buprenorphine (33.3%). In 95% of cases, users related this evolution to the unavailability or financial inaccessibility of the products they consumed in their home countries. In 3.1% of cases, they said they wished to replace former products with other, more attractive ones, or simply that they wanted a change. There is a very pragmatic adaptation to the availability of products on the local market to quench a state of craving, as Olga explains: “I arrived here at full craving. For vint. [...] I met a few Russians and Poles. I learned from one of them about subutex^12^ [buprenorphine], they told me it was like heroin, that it helped with craving. [...] At first, I snorted it. It helped me; the craving went away. [...] Then I started injecting it, as a habit. When I started, I started losing the use of my arms and legs [...] Then they told me about skenan^13^. That it doesn’t destroy you. So I switched over to sknenan.” (Olga, 27 years old, Russian).

#### Evolution of equipment sharing practices

After they arrived in France, people who use drugs became more aware of viral contamination risks (HIV and hepatitis C, particularly) through syringe sharing. Although 49.9% said they had already shared a syringe in their home country, the proportions shrink to 13.9% after they arrived in France and to 9.3% in the month before the study, which is a lower rate compared to the French-speaking PWUD, 26% of whom reported that they engage in syringe sharing [18]. Some users did know about infectious risk related to equipment sharing before migrating, but their testimony shows that some users did not know they carried a virus for lack of testing (“They asked me if I had hepatitis, and I always said no because I didn’t know I had it at the time,” Alik, 34 years old, Russian born in Chechnya) and that a discourses about willful transmission continues to gain traction (“There is a 60 to 70% greater chance [than that of contamination through used syringes] that someone who has a nasty disease wants to contaminate someone next to him who is clean,” Sarkis, 39 years old, Armenian).

### Family, employment, and prison (Table 5)

#### Family context, 16–18 year olds

The interviews reveal chaotic life trajectories featuring complex familial configurations that make social ties unpredictable at home and that destabilize future prospects. Users’ life stories often include distant relationships, whether because of childhood abandonment (“I don’t have anyone. My mother left me, I lived with my father, and then when I was seven or eight years old, my father left me too, [and] some neighbors helped me out,” Lévon, 27 years old, Georgian born in Armenia) or because of members of their family rejected them (“My father was a colonel and he was killed when I was 17. [...] Three years later, my mother died. [...] First I went to Belarus, where my mother was from. Her older sister was there. [...] When I got there, she said ‘there you go, that had to happen, it’s just like your mother to get into trouble: travelling around and getting married to a soldier [...] and then here you are, butt-naked, no money and asking for help. What do I have to give you now?’” Ulan, 43 years old, Russian born in Kazakhstan).

At age 16, more than 10% of survey respondents no longer lived at home (3.6% lived with a family member and 6.6% lived outside any family support: alone, in a relationship, at school, in boarding school, in prison, or at the house of a third party). At age 18, three in four had already slept either in a shelter (72.7%) or on the street (79.2%), a sign of their precarious life conditions and of the fragmented family structures that might have provided stable housing.

#### Current relationship status

Six in ten survey respondents (60.9%) said they were in a relationship at the time of the study. Among them, 62.7% said they live with their partner permanently, 23.2% said they did not live with a partner, and 14.1% said they sometimes did. Although some are married and have children (who either came along or were born after they migrated), they also described events of breaking family ties, such as the sudden departure of their spouse and the loss of custody of their children, as Dimitri explains: “I was under heavy stress because I was going through a divorce and I lost custody of my child. [...] I asked my ex-wife, I sent her money many times, but they don’t let me see my son. She got remarried and [...] she told me I would never see [him] again.” (Dimitri, 29 years old, Russian). Although these breakups mostly happened before migration in sometimes complex situations (“My dad slept with my wife, they pulled a real fast one on me. I became homeless, so yeah... very stressful. My younger brother had decided to jump on the opportunity of my divorce, because I was so out of it, to swindle my mom and to grab the apartment illegally. He decided to sell it to start a company with our cousin. At the end of the day, my mom rejected me, so that shook things up even more,” Dimitri, 29 years old, Russia), some happened in France (“I got divorced in 2009. I was married to a Russian who came here with me. She got remarried with a French guy. My son is 12 years old and he stayed with her,” Nikolaï, 39 years old, Russian).

#### Education level and employment status in home country and in France

Although they have sometimes pursued high levels of studies (43.0% have some higher education) and held important jobs in their home country, almost all (96.8%) respondents are now in precarious social positions, either unemployed or holding several undeclared and risky jobs. The high proportion of respondents who underwent a loss of social status said it happened either before or after they migrated.

Although some users were part of the upper socio-cultural class and earned significant salaries in their home country, a few explained that they burned through all of their savings, either because of a gambling addiction (“I got married at City Hall, I even created two companies and I was starting to make a good living. [...] Then they got these gambling machines in Samara. And I let myself get caught up in there, I lost the first sum of money. Which came from my marriage. Then I went to live with my father,” Dimitri, 29 years old, Russian) or because of narcotics use (“I was a production engineer […] I did my practical internship in a plant, and then I stayed to make more money. […] [I left] because in the last years I was already starting… actually drugs started to cause a lot of trouble for me,” Youri, 28 years old, Russian).

The majority of respondents who are unemployed (83.1%) said that they are not authorized to work in the country where they arrived, because they do not have the required documentation (residence permit, work permit). They then discuss their precarious conditions and the risk of not getting paid if they except illegal work: “I am undocumented, so no one gives me work. For unregistered work, they take you for one month and then they let you go.” (Anzor, 34 years old, Born in Chechnya). Other unemployed respondents are either disabled (0.3%), or more often, are registered as unemployed (16.7%). Although some have registered administratively to appear lists of jobseekers in France, some like Mosvar explain how difficult it is to claim your rights when you do not speak the language: “I was taken off the list at Pôle Emploi [the French unemployment office]. I don’t speak French, so I found someone to come with me so that I can register. They took me in and then it took me off again after one month. I had to do everything all over again. No one wanted to come with me. I didn’t get why they have taken the off, and on again, and off again, and on again, and off again.” (Mosvar, 39 years old, Chechen born in the Ingush Republic).

These trajectories, in which people who use drugs lose social status (sometimes experiencing a strong sense of injustice) lead some to reflect on their life stories, like Sarkis, a 39-year-old Armenian who wonders how this loss of status could have happened: “I was a professional dancer, I graduated from the Armenian School of Choreography, […] I had a good life, and when I was doing well I always helped others, just like I do now. Even now in the wheelchair, whether or not I can, I share my leftovers with anybody. […] I drink vodka, champagne, cognac, I ate caviar, and in my family there’s everything, everything, everything. Now I’m here and... I don’t know how […] I am morally exhausted, exhausted by this inversion. This coldness… The cold, it doesn’t even affect my body, do you understand? I am hungry because of the cold, I can’t even eat correctly.” (Sarkis, 39 years old, Armenian).

#### Prison records

Six in 10 respondents (57.6%) said they had spent time in prison (with diverse sentences for 61.5% or about two-thirds of them). Although most incarcerations happened in their home country (59.7%), others were given either in countries that they crossed before arriving in Paris (35.5%) or/and France (28.7%).

### Life conditions in France (Table 6)

#### French language proficiency and assistance

Almost 4 in 10 respondents (37.6%) have no knowledge of French and cannot communicate; 41.4% know a few expressions that allow them to communicate every so often, and only one in five (20.9%) consider themselves to be completely autonomous, meaning that they are able to interact in French in a large diversity of daily situations. Among those who do not speak French, almost 6 out of 10 (56.4%) can count on the help of friends or family either exceptionally (17.2%) or regularly (39.2%): this leaves more than 4 in 10 (43.6%) without any linguistic assistance despite their inability to speak French.

#### Financial resources

Most respondents said they live on donations from family/friend (47.6%, almost half), odd jobs (36.2%), various benefits (23.0%), theft (17.7%), and/or begging (5.8%). The interviews reveal tensions between work (often unregistered), various benefits, petty crime, and begging and show that some users organize their time according to the opportunities of financial survival that come up for them: “I don’t have the possibility of exercising my profession here, musician or whatever, so that’s all the worse for them, I’ll be a cleaning lady, but it’ll be better to work honestly, I am tired, I don’t want to steal, I don’t like it, even though I was quite good at it. But it’s wrong, shameful...” (Olga, 27 years old, Russian); “I haven’t worked in 6 months. [...] I live on the holy spirit. Sometimes I steal, sometimes I beg... Sometimes they help me out.” (Nikolaï, 39 years old, Russian); “I go to Saint-Eustache Church at 8 am, where there’s a soup kitchen, and in the morning, I go to 110 Les Halles to have breakfast. I come here, they give me milk, and I spend the rest of the time at the library. I don’t have a family or anything, so I don’t need any money.” (Ulan, 43 years old, Russian born in Kazakhstan).

#### Housing

Survey respondents often described precarious housing conditions that evolve according to outside events. For the coming night, only 16.2% (1 in 6) had personal housing (a hotel room, or more rarely, an apartment that they rent partly through their own means by working). The great majority of them (83.8%, or 5 in 6) planned to sleep either at the house of a third party (14.7% of respondents), at a shelter (40.5%), or in a location not designed for housing (squat, vehicle, public space) (28.6% of respondents). The following quotes illustrate the difficulties of this last group, who makes up about a third of respondents: “Currently, I wander with my luggage: a tent, two sleeping bags... So it’s a heavy bag, I wear a special corset to make it lighter. 30 kilos on my back, same as a handcart.” (Dimitri, 29 years old, Russian); “[These days, I live] in an abandoned house, a squat. Six people live there, Chechens and Georgians. Only Russian speakers.” (Austin, 45 years old, Russian born in Georgia); “Four months ago, the 115 [a social emergency public service] and France Terre d’Asile [association advocating for the rights of migrants] found me a hotel room. My friend asked me to host an acquaintance of his who lived on the street. I hosted him for three weeks and the director of France Terre d’Asile learned about it, and they kicked me out of the hotel. They closed my file and I was on the street [again].” (Sarkis, 39 years old, Armenian).

Although the great majority of respondents (90.0%) lived in precarious housing at the time of the study (collective or individual housing provided by associations^14^, houses of friends or family, hotel rooms on their own expenses) or even very precarious housing (squat, street, vehicle) (28.6%), some interviewees, like Youri, described how difficult it was to get help for administrative tasks, even from social organizations: “In terms of health and getting medical help, they can do something, but not for anything else. Finding me somewhere to sleep doesn’t interest them. I only asked them to call somewhere to give them an address and a phone number, and they said they couldn’t, that it wasn’t their job and that it’s not up to them.” (Youri, 28 years old, Russian). Others like Olga described the tough life conditions in shelters: “We ended up at the Sleep In, which is shit. Kaspar couldn’t even do it, he went and slept on the street. He was hitting everybody, if only you knew... It’s impossible to be there. It’s a mess, with the druggies, the dirt, the whores, the stink, bozos, losers, psychos, some shooting up and throwing up and fucking everywhere, it’s a mess, everything is full of shit and spit [...] it’s shit. Boys sleep on one side, girls on the other; there are two showers for everybody, so you have to be careful not to catch anything. They serve food at 7 am. He lived there for a month and then he couldn’t. He went back to the street. It was winter. I went back there very reluctantly. I would see him and then I went back to the Sleep In. [...] And then I couldn’t do it anymore either, so I went with him in the street.” (Olga, 27 years old, Russian).

#### Documentation in France

It is clear from the interviews that the great majority of interviewees are waiting for the legalization of their administrative situation. Acquiring legal documentation is considered to be very important, especially as users often see it as a compulsory first step in acquiring housing, work, and treatment without risking expulsion, as Jino explains: “I need treatment, but first I need to settle in with the documentation, I need to know if I’m here or there, so that I don’t get controlled tomorrow and suddenly exported somewhere else.” (Jino, 23 years old, Polish born in Georgia). In this context, requesting hepatitis C treatment is sometimes described by those who carry the disease as a way of acquiring housing more than as an end in itself (“Someone told me that you could get housing for little while here in France if they give you treatment for hepatitis,” Dimitri, 29 years old, Russia).

## Discussion

### A particularly vulnerable population

Our study highlights the extreme vulnerability of people who use drugs who migrate from Eastern Europe to France. This vulnerability translates into a fragile position regarding both their personal trajectory (traumatic experiences in their home country, family breakups, loss of social status) and their living conditions (very precarious housing, low access to employment). Compared to other drug users (French-speaking or not) from similar services, they display more precariousness, which is clear from their difficult housing situation and by more feeble prospects of integrating socio-economically [12, 13, 19, 20]. Migration experiences and life conditions in the host country are often associated with a high level of mental distress (stress, anxiety, depression), as was observed among migrants from sub-Saharan Africa in the ANRS-Parcours study^15^ [21].

Even if the decision to leave one’s country is always difficult in contexts of forced migration, some researchers have remarked that those who see it through stand out both in socio-cultural standing (compared to other users in walk-in services, which has been observed in France [13] and in Germany [22]) and in their hopes of gaining access to adequate treatment [12, 19, 23]. With a higher proportion of people who have some higher education than among French-speaking PWID (43.0% vs 27.6%) [13] and a significant focus on gaining access to treatment and a normalized lifestyle, our observations confirm these findings. Drug users often see France as an open and “human” country, a mirror image to their experience before migrating. Access to free, anonymous addiction healthcare, and harm reduction services (not requiring medical insurance and administrative registrations) contribute to this positive perception of France as a host country.

### Improving care in France and in drug users’ home countries

One of our study’s results is that Russian-speaking drug users adhere to infectious risk prevention measures recommended by harm reduction practices when they are given the material means (access to sterile equipment) to do so, like they are in France: (1) they share syringes less frequently than before migrating (13.9% of cases in the last month vs 49.9% in their home country); (2) the sharing rate noted in the last month is twice as low as that observed among French-speaking PWID (26%), even though the latter are less exposed to equipment sharing (38% of syringe exchange in lifetime) [18]. These results encourage us to make two recommendations: consolidate global care in France (both healthcare and social care) for this particular population and raise awareness among political leaders of their respective home countries to evolve from repressive policies to ones focused on prevention and treatment.

Concerning the first point, beyond implementing targeted measures (like medical testing and antiviral treatment) [22], some French language researchers recommend hiring interpreters at walk-in services [12, 24] and taking into account cultural variables when offering already existing care [14, 23] in order to facilitate follow-up. The goal is to reduce the number of linguistic and cultural obstacles further marginalizing an already wary population, all the while meeting the most isolated individuals where they are through peer outreach aimed at encouraging the “creation of social ties” that could gradually bring them to seek treatment [23–26]. In that context, one of our study’s limitations is to have focused only on individuals who have already taken the initiative to seek out healthcare centers and harm reduction services. It does not give us any information of users existing outside these services (meaning about active users who do not attend specialized facilities or about those who have stopped using after migrating without receiving substitution treatments), which could open new research paths and perspectives.

Concerning the second point, it is a matter of international “social solidarity” [14] to contribute to a situation in which these individuals receive adequate treatment not only in France but also in their home country. This is particularly crucial for countries like Georgia (Georgians make up 57.2%, or more than half, of our sample for this study) where harm reduction programs are evolving, particularly following the example of Médecins du Monde [3, 8] of the Global Funds to fight AIDS, Tuberculosis, and Malaria [27]. Local authors who have proven the feasibility and efficacy of prevention measures (particularly when it comes to HIV transmission) insist on three aspects: the costs that these countries with limited financial abilities have to bear [28], the absolute necessity of involving women in these social programs [29], and the difficulty of implementing long-term measures in a context of continuously enforced repressive policies [10], which suggests that we should make use of international political levers [3].

## Conclusions

This study based on mixed methods gives us better knowledge of the sociodemographic profiles and migration trajectories of drug users coming from Eastern Europe and living in Paris. Beyond epidemiological objectivation, our qualitative analysis highlights the day-to-day difficulties of this population and their high vulnerability, no matter their country of origin or reasons for migrating (be they health-related, economic, ethnic, or political). The results of our study lead us to make two recommendations: to offer a more comprehensive access care to migrant drug users in Paris and to help activists to include a stronger public health approach in drug policies in Eastern Europe.

## Data Availability

The datasets generated and/or analyzed during the current study are not publicly available due to requirements from the committee for the protection of individuals in Créteil.

## Abbreviations

PWID: People who inject drugs
HIV: Human immunodeficiency virus
ANRS: Agence Nationale de Recherches sur le Sida et les hépatites virales
CAARUD: Centre d’Accueil et d’Accompagnement à la Réduction des Risques pour Usagers de Drogues
CSAPA: Centre de Soins, d'Accompagnement et de Prévention en Addictologie
AME: Aide Médicale d’Etat
CMU: Couverture Maladie Universelle
MDPH: Maison Départementale des Personnes Handicapées

## Footnotes

1 Tourism or work visa.

2 *Cherniashka* is a mixture of opium poppy straw and other chemicals.

3 *Buprenorphine* is an opioid medication, which contains a morphine-like substance.

4 *Desomorphine* is a synthetic morphine.

5 *Vint* is a mixture of methamphetamine and pseudoephedrine.

6 *Krokodil* is a derivative of codeine.

7 *Pervitin* is a methamphetamine.

8 *Ephedrine* is a sympathomimetic amine and substituted amphetamine.

9 *Jeff* is a monoamine alkaloid and psychoactive stimulant.

10 C*rystal meth* is one form of the *drug “*Methamphetamine” (a synthetic psychostimulant close to decongestants such as ephedrine).

11 Subutex and Temgesic.

12 *Subutex* is an opioid substitution therapy that contains buprenorphine (a morphine-like substance).

13 *Skenan* is a powerful painkiller that contains morphine.

14 In structures open to the general public (associative apartments, collective housing, associative hotels rented by emergency social services) or specially designed for people who use drugs (post-cure establishments, therapeutic apartments, housing centers), which are payed for or managed by associations.

15 Whether because of threats received in their home country or because of their illegal activities in their host country.
